# Supplementary material for: Unraveling Dengue Virus Diversity in Asia: An Epidemiological Study through Genetic Sequences and Phylogenetic Analysis
Source: Viruses. 2024 Jun 28;16(7):1046. doi: 10.3390/v16071046 (PMC11281397; doi:10.3390/v16071046)

Figure S3A. DENV-3I Clade 2 subtree

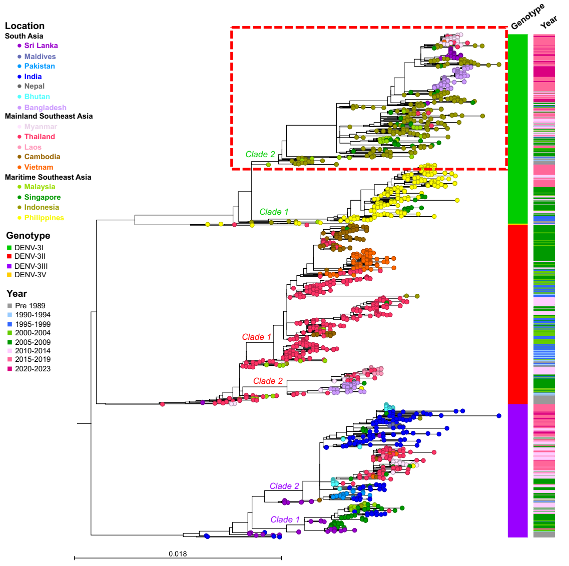

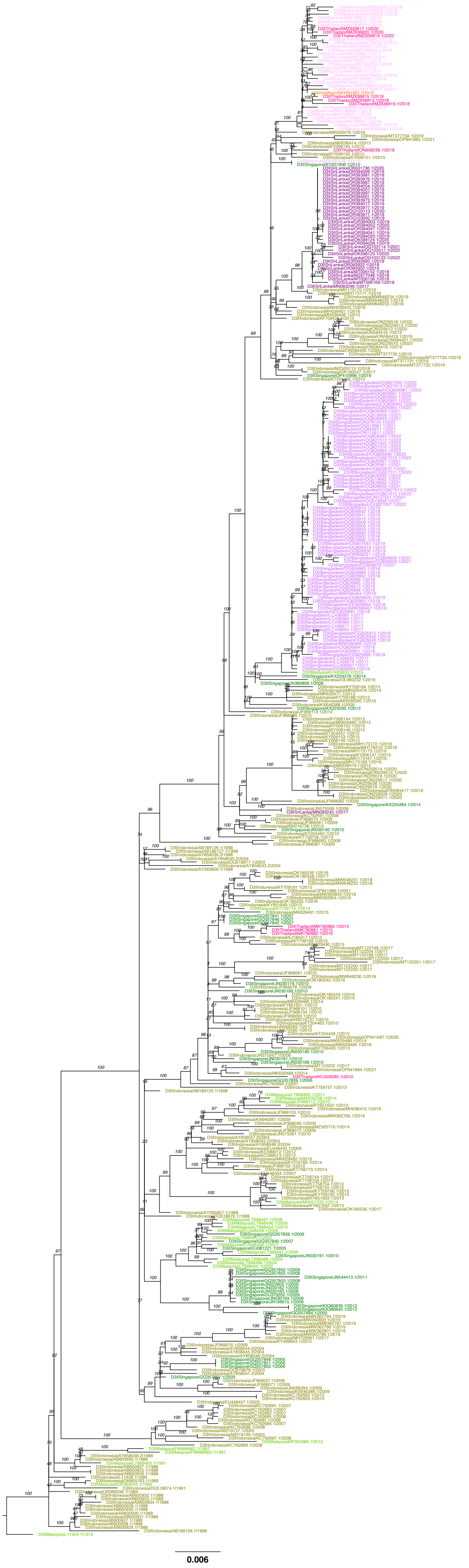

Figure S3B. DENV-3I basal and clade 1 subtree

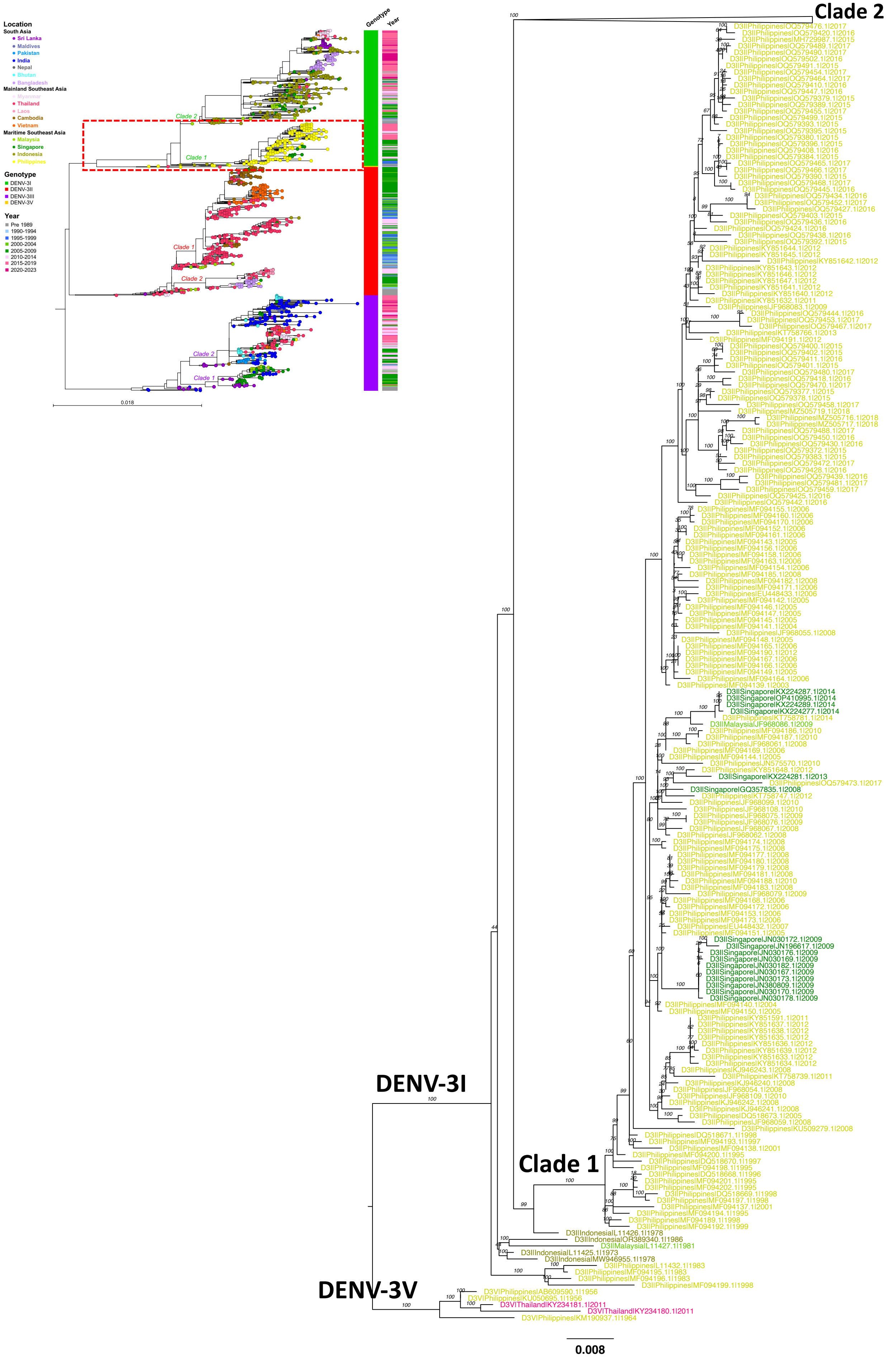

Figure S3C. DENV-3II subtree

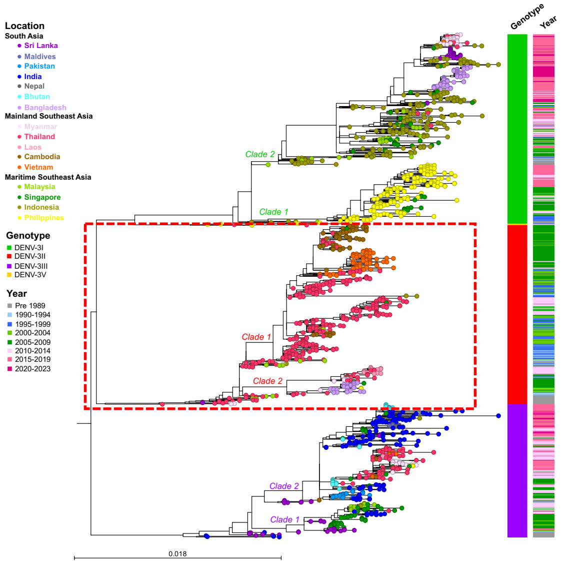

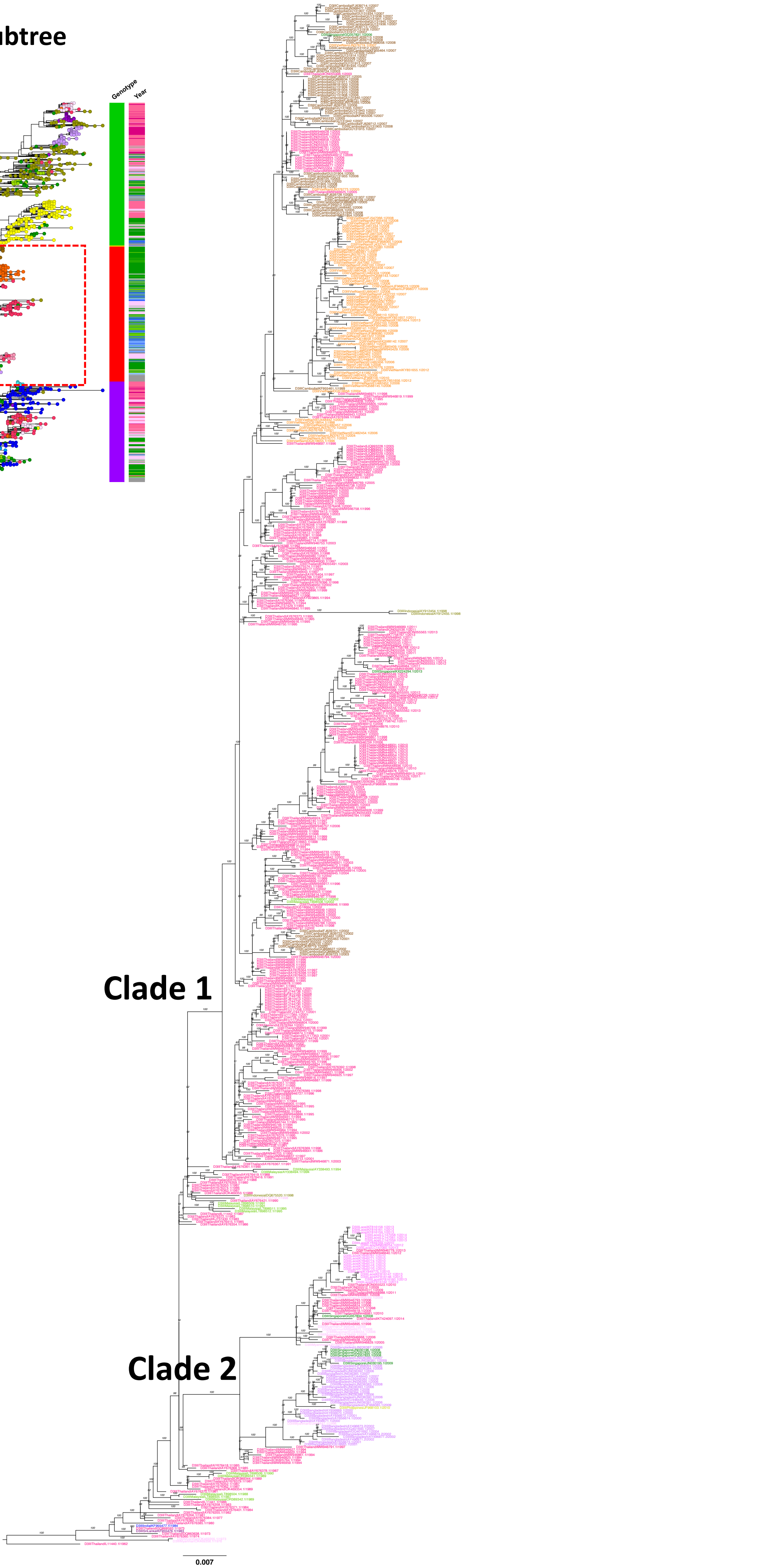

Figure S3D. DENV-3III subtree

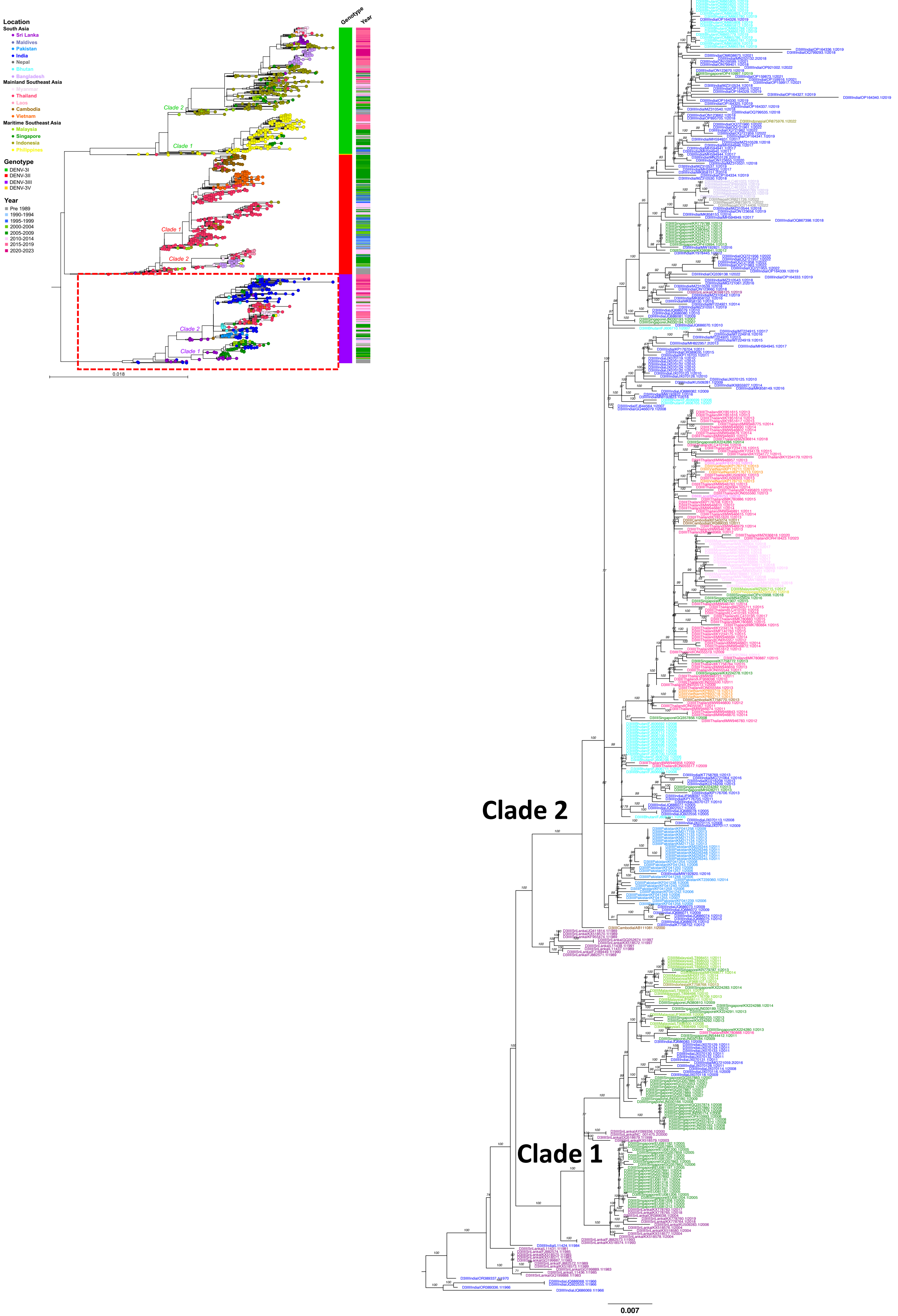

Supplement: Supplementary file 1 [file viruses-16-01046-s001.zip › Figure S3.pdf]
