# Supplementary material for: Unraveling Dengue Virus Diversity in Asia: An Epidemiological Study through Genetic Sequences and Phylogenetic Analysis
Source: Viruses. 2024 Jun 28;16(7):1046. doi: 10.3390/v16071046 (PMC11281397; doi:10.3390/v16071046)

Figure S4A. DENV-4I Clade 3 (upper) subtree

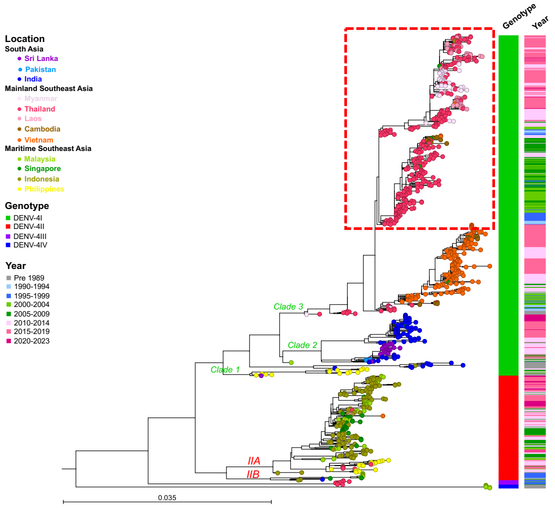

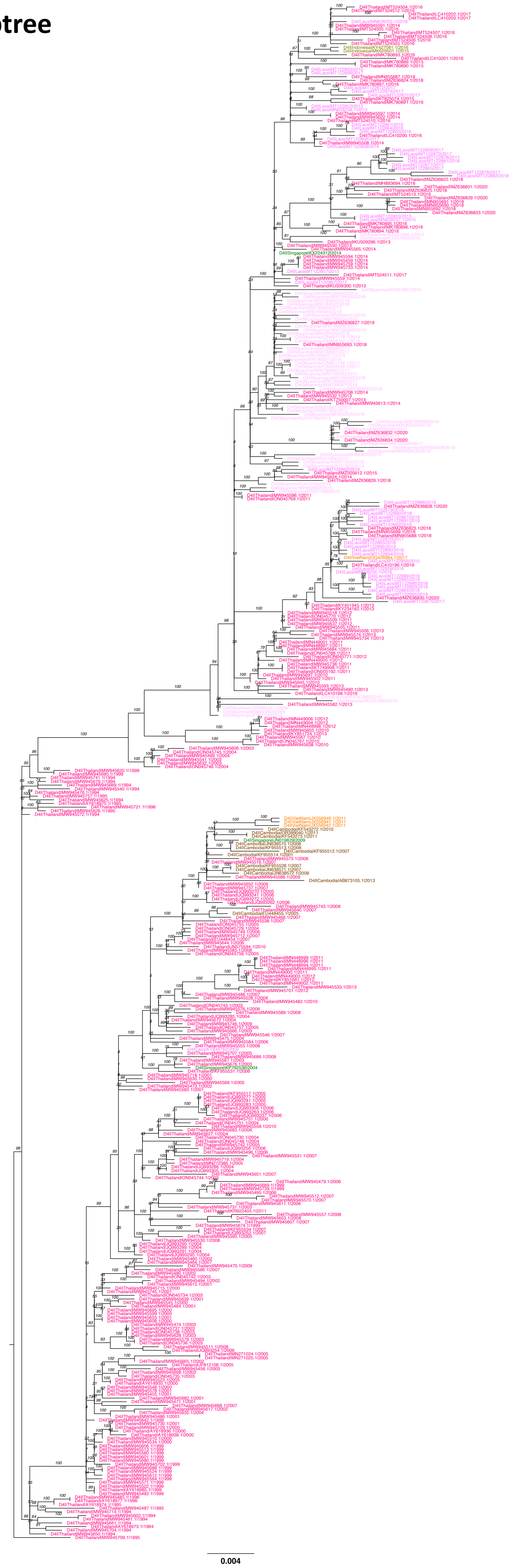

Figure S4B. DENV-4I Clade 3 (lower) subtree

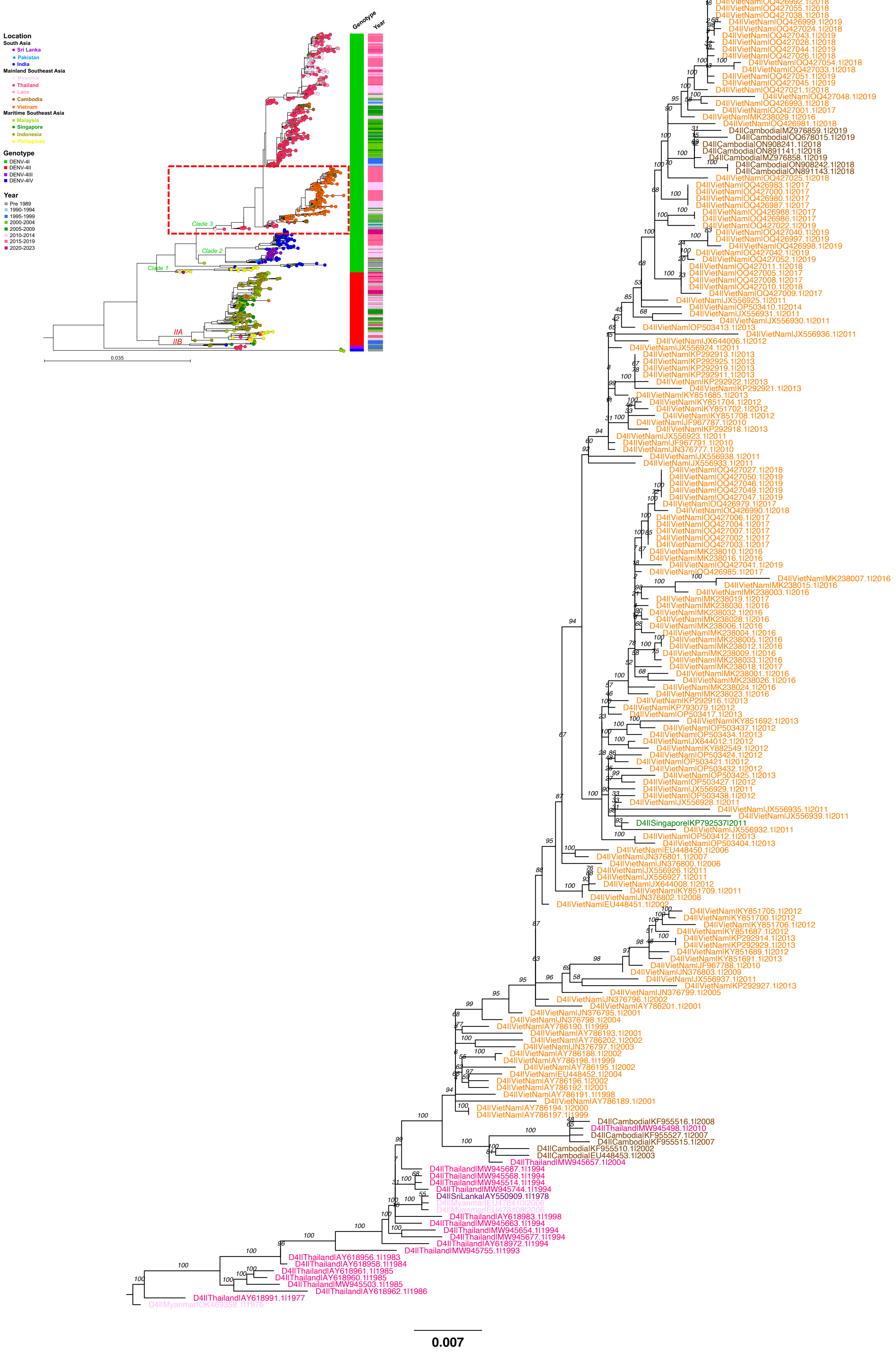

Figure S4C. DENV-4I Clade 1, 2 subtree

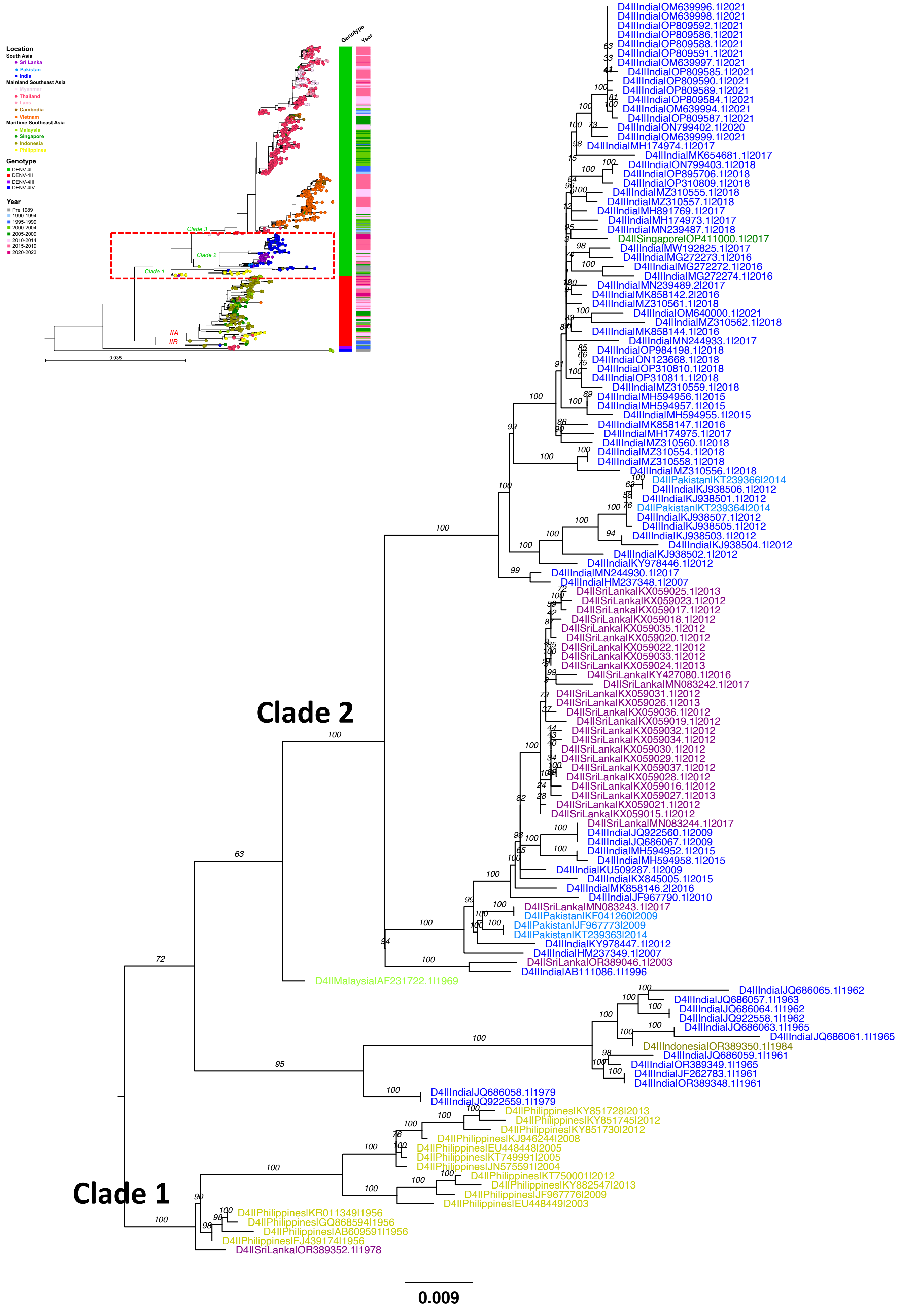

Figure S4D. DENV-4II, 4III, 4IV subtree

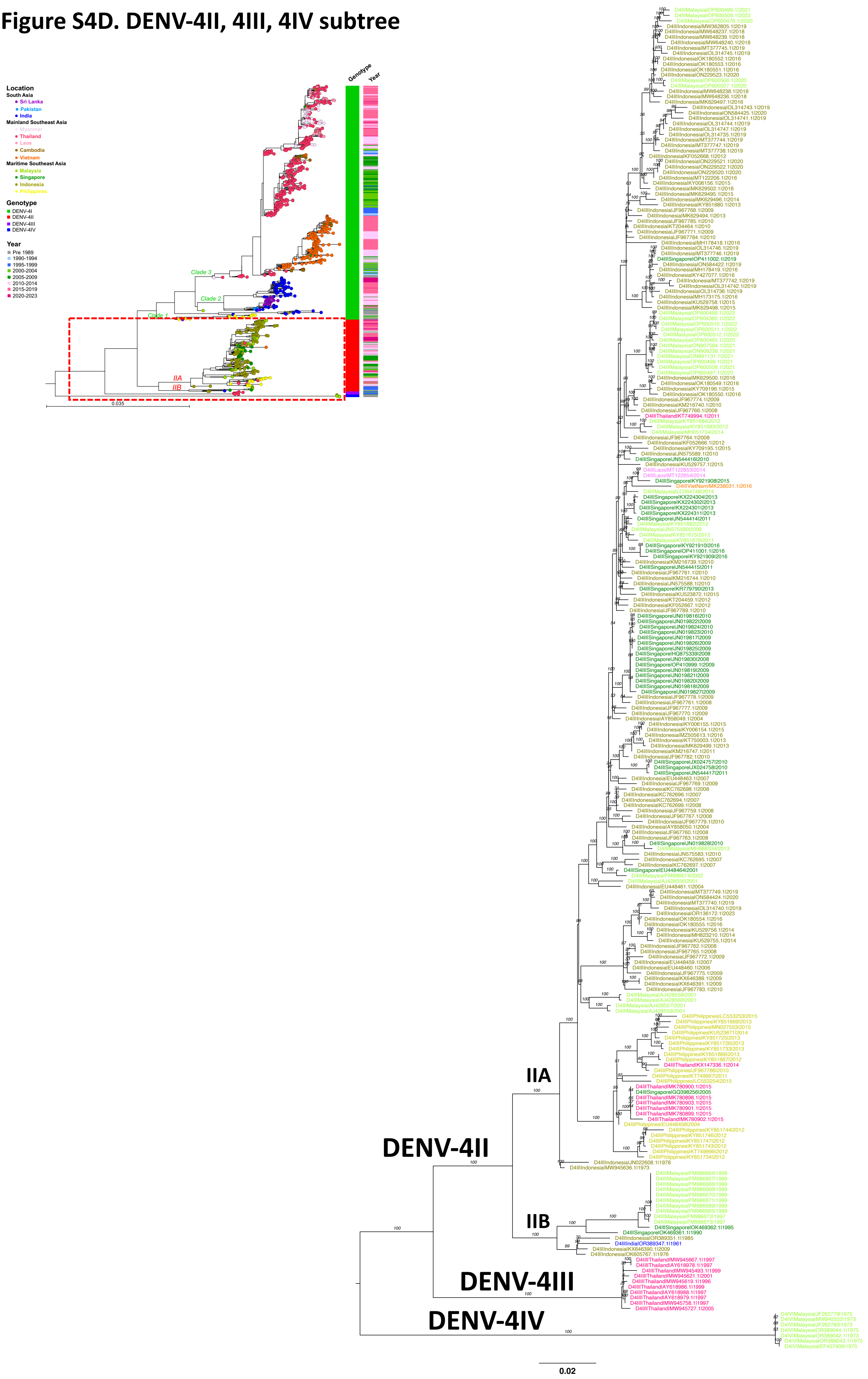

Supplement: Supplementary file 1 [file viruses-16-01046-s001.zip › Figure S4.pdf]
