# Supplementary material for: Unraveling Dengue Virus Diversity in Asia: An Epidemiological Study through Genetic Sequences and Phylogenetic Analysis
Source: Viruses. 2024 Jun 28;16(7):1046. doi: 10.3390/v16071046 (PMC11281397; doi:10.3390/v16071046)

Figure S2A. DENV-2II Lineage 2 (upper) subtree

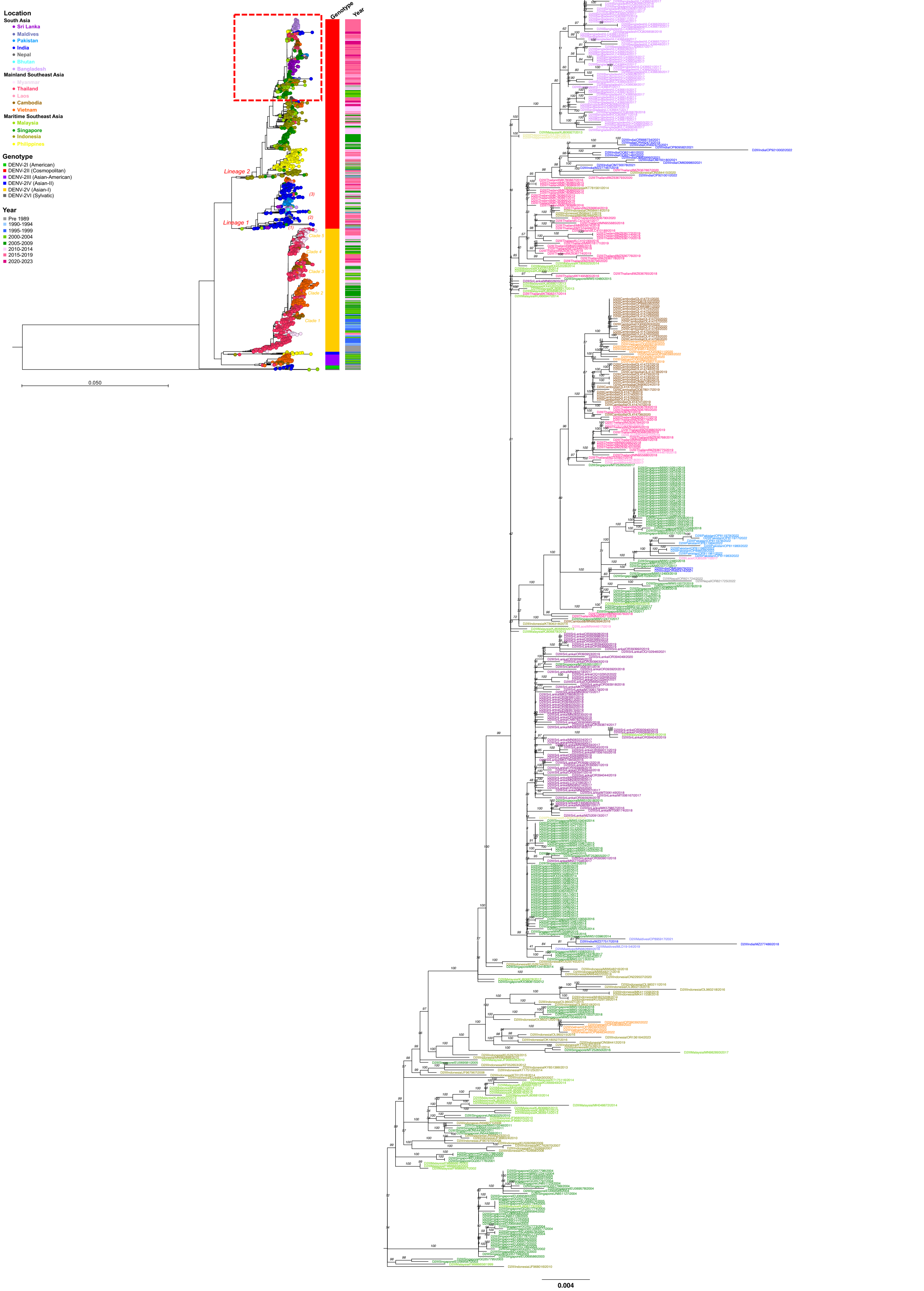

Figure S2B. DENV-2II Lineage 2 (middle) subtree

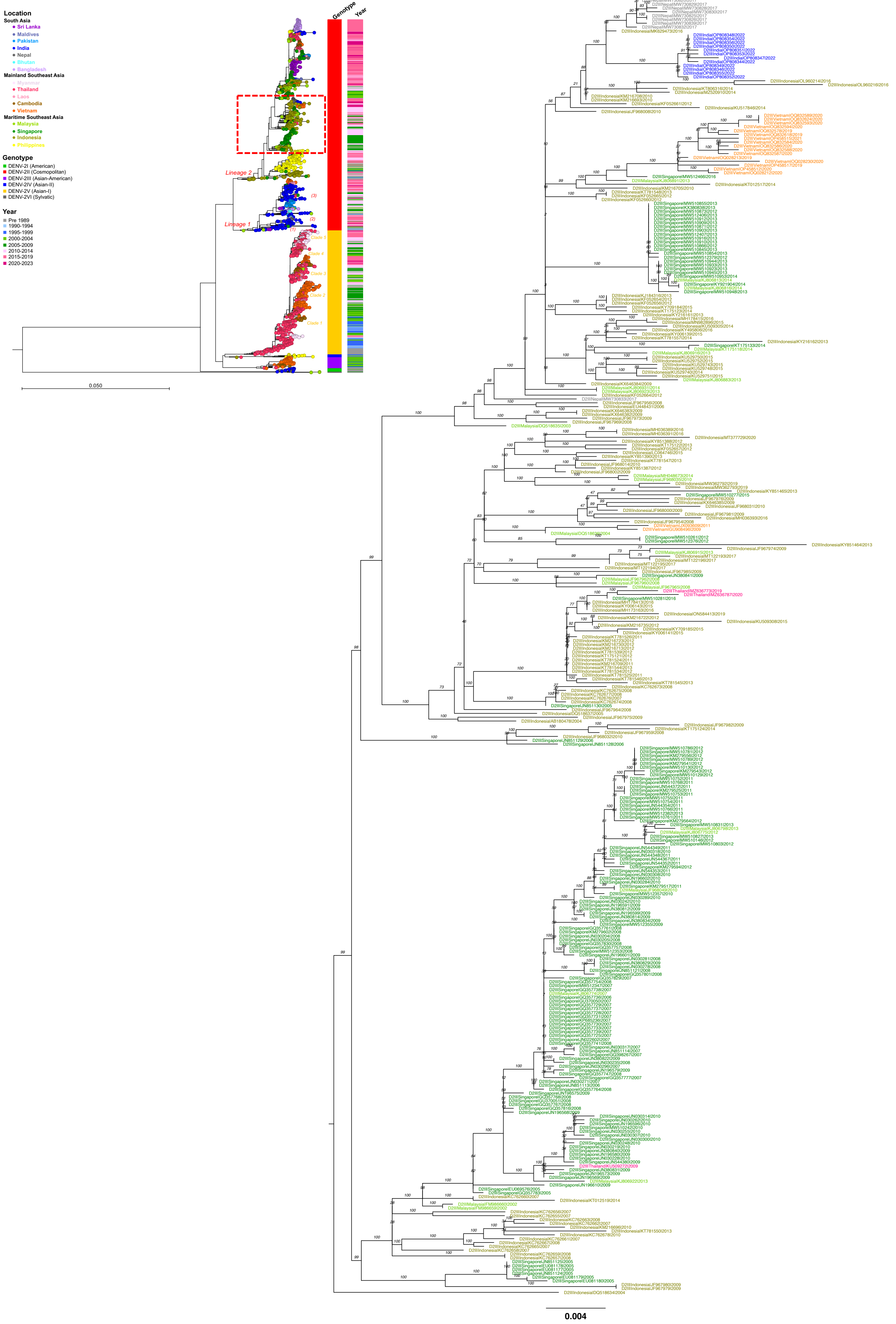

Figure S2C. DENV-2II Lineage 2 (lower) subtree

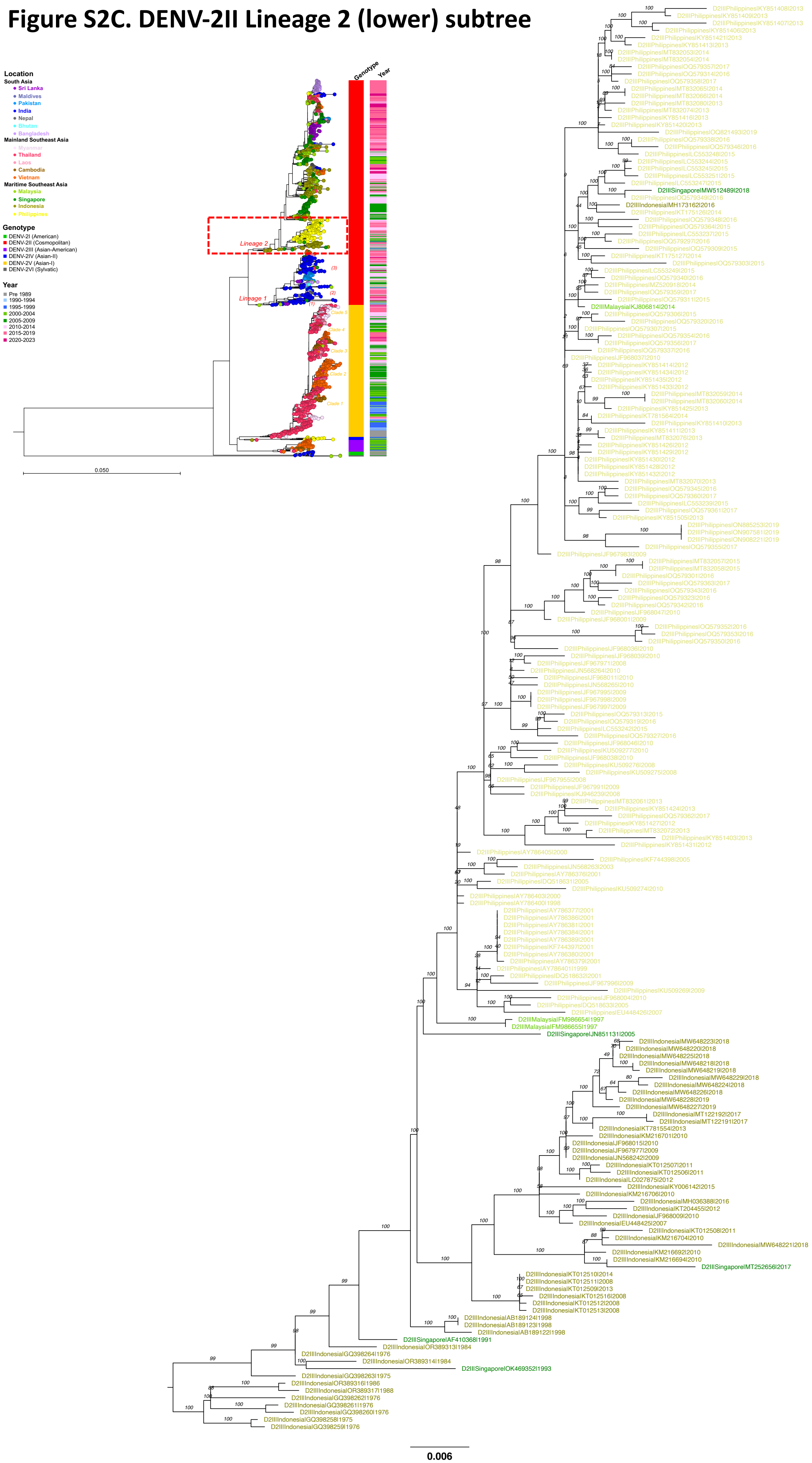

Figure S2D. DENV-2II Lineage 1 subtree

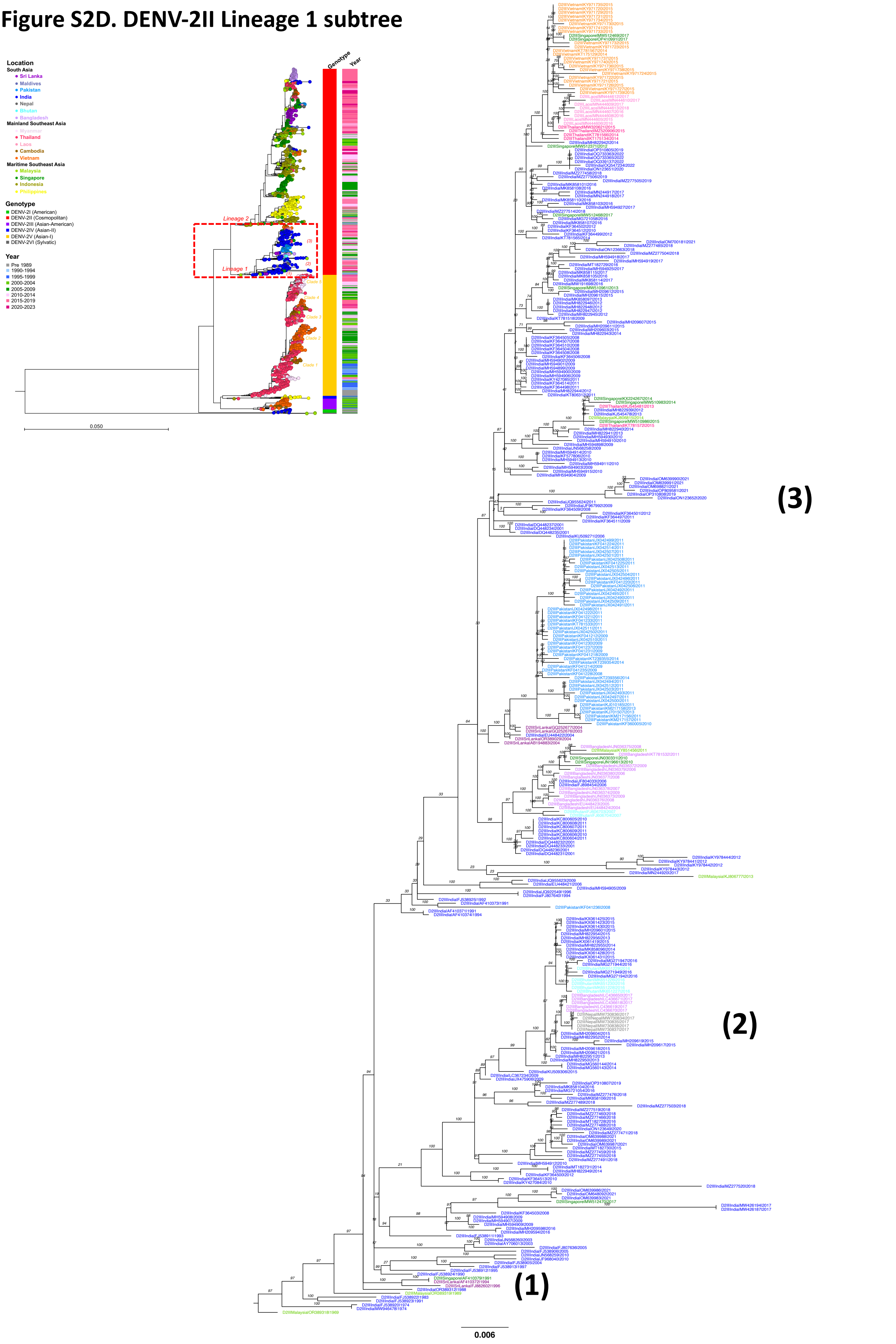

Figure S2E. DENV-2V Clade 3, 4, 5 subtree

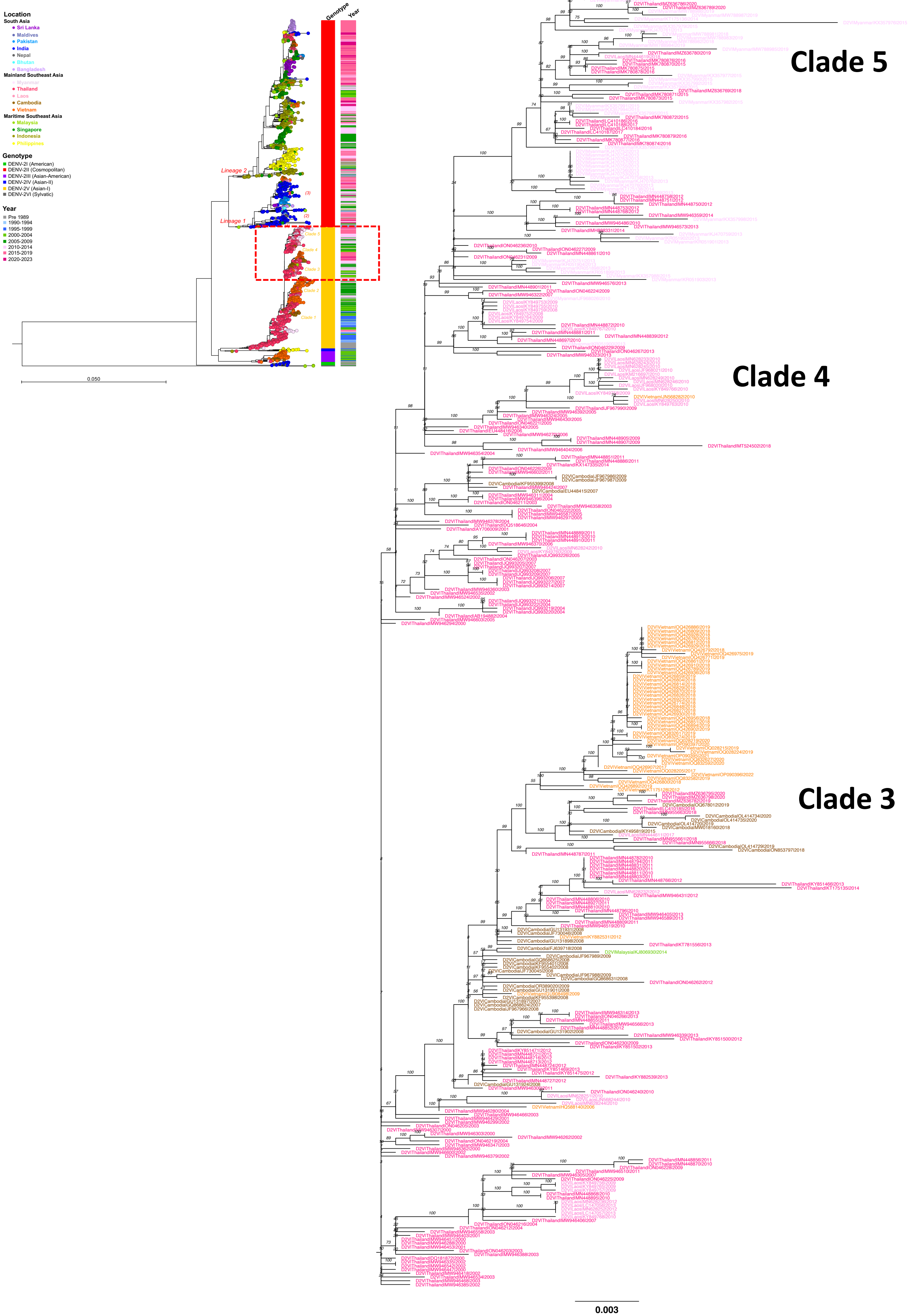

Figure S2F. DENV-2V Clade 1, 2 subtree

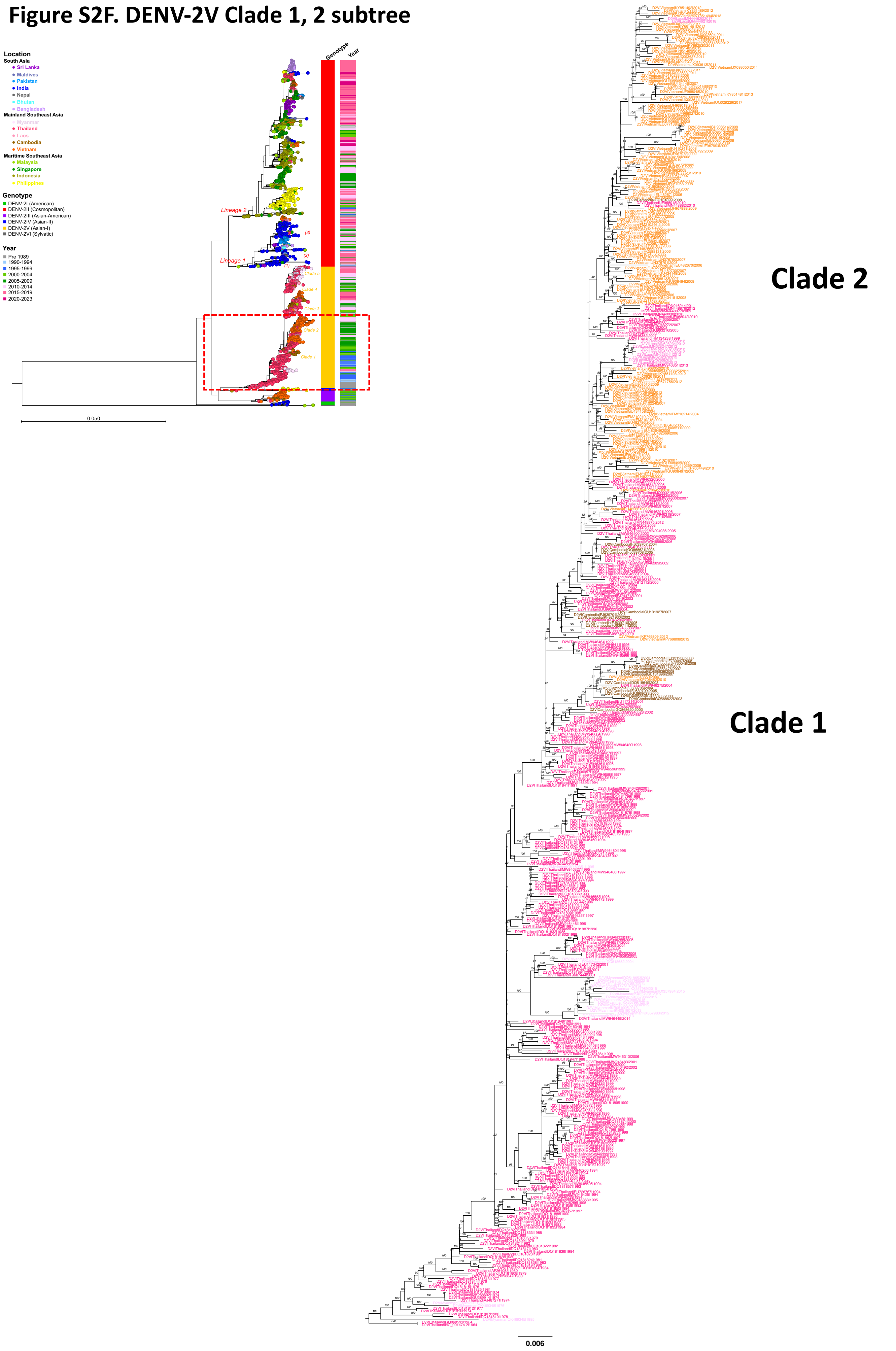

Figure S2G. DENV-2I, 2III, 2IV, 2VI subtree

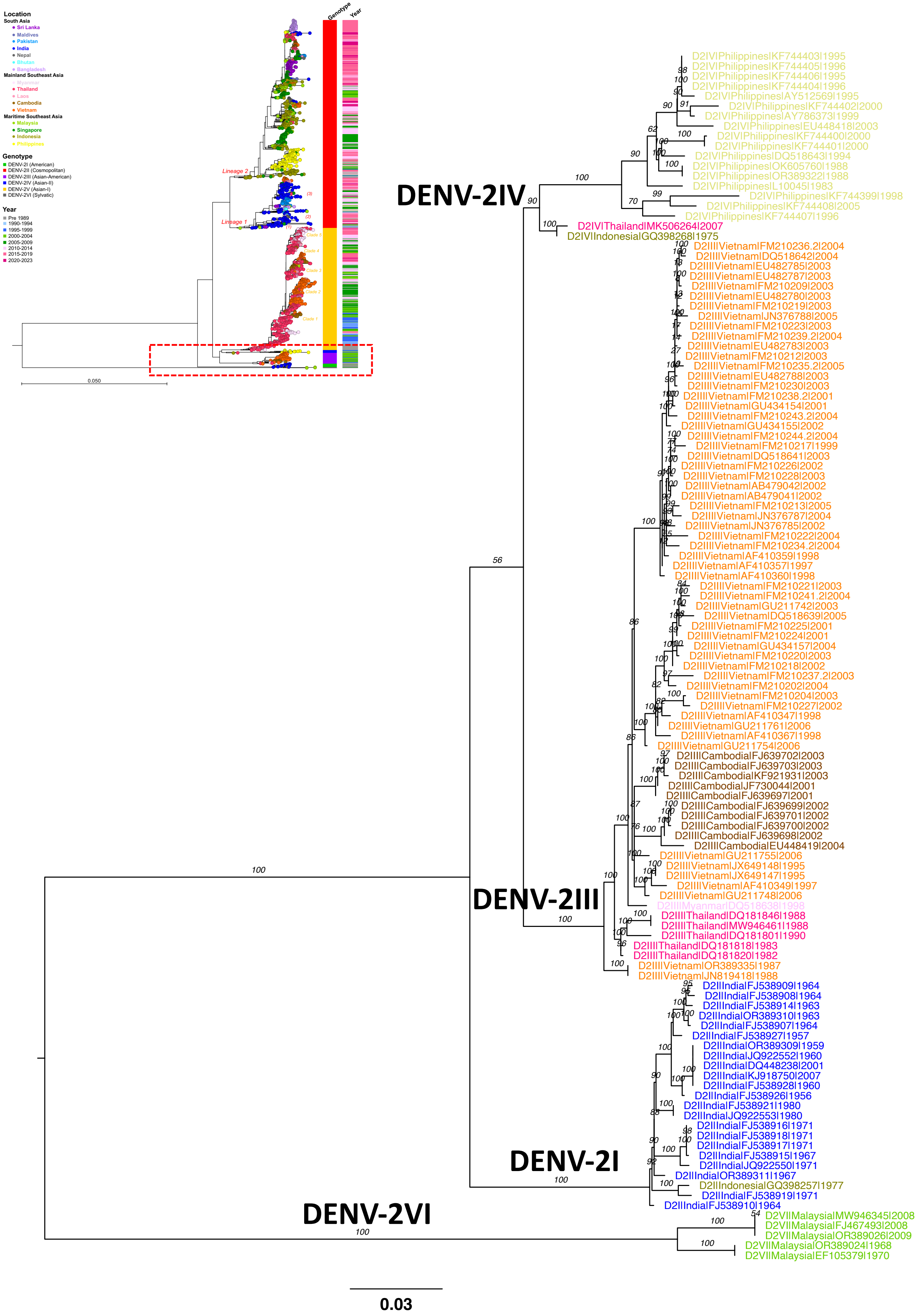

Supplement: Supplementary file 1 [file viruses-16-01046-s001.zip › Figure S2.pdf]
