# Supplementary material for: Unraveling Dengue Virus Diversity in Asia: An Epidemiological Study through Genetic Sequences and Phylogenetic Analysis
Source: Viruses. 2024 Jun 28;16(7):1046. doi: 10.3390/v16071046 (PMC11281397; doi:10.3390/v16071046)

Figure S1A. DENV-1I Clade 3 (upper) subtree

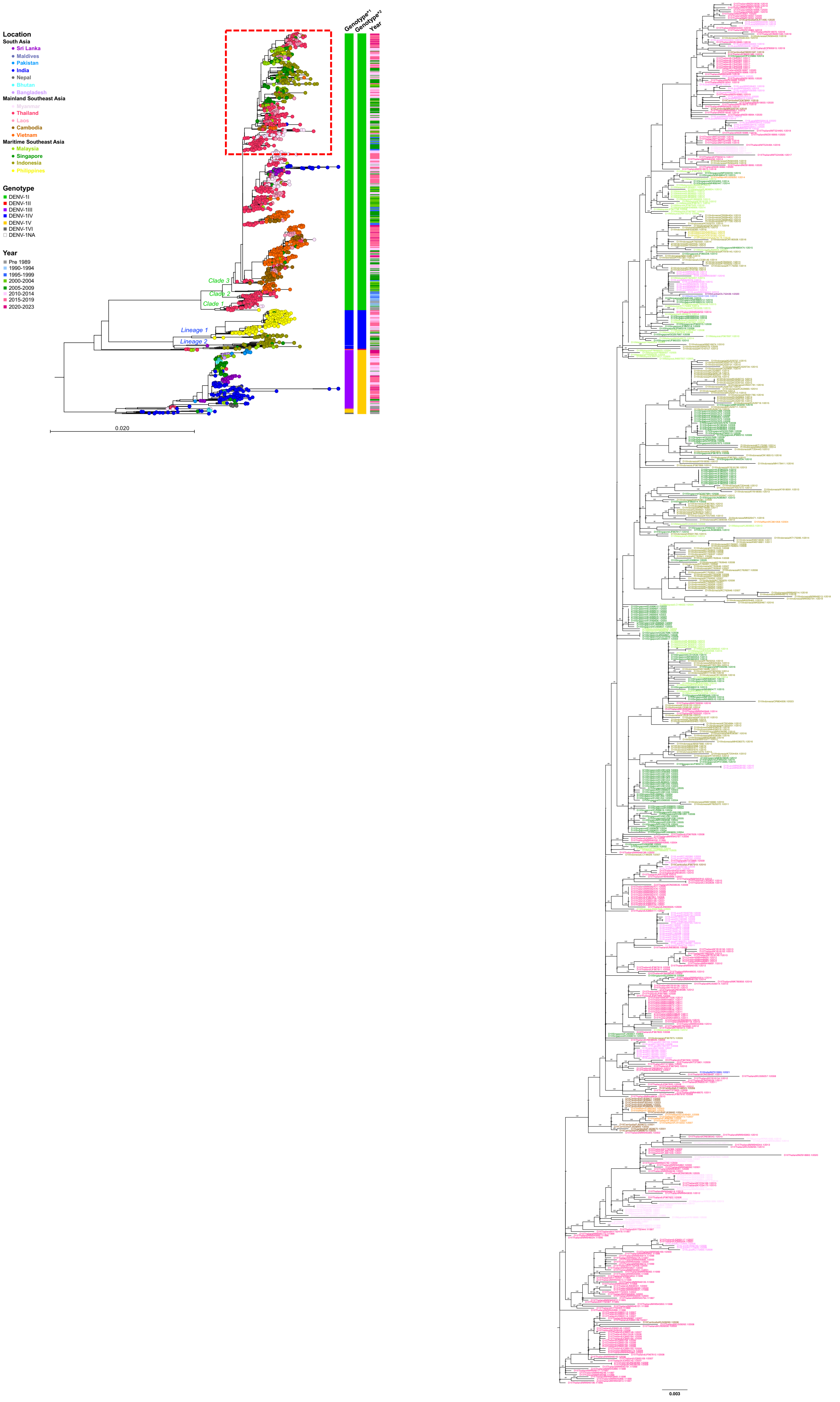

Figure S1B. DENV-1I Clade 3 (middle) subtree

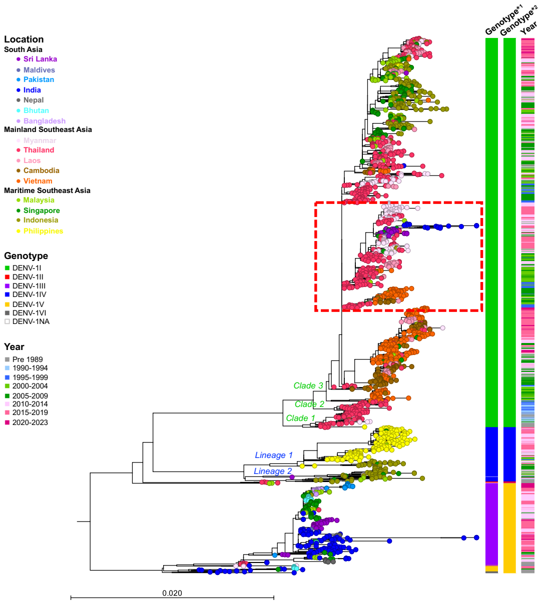

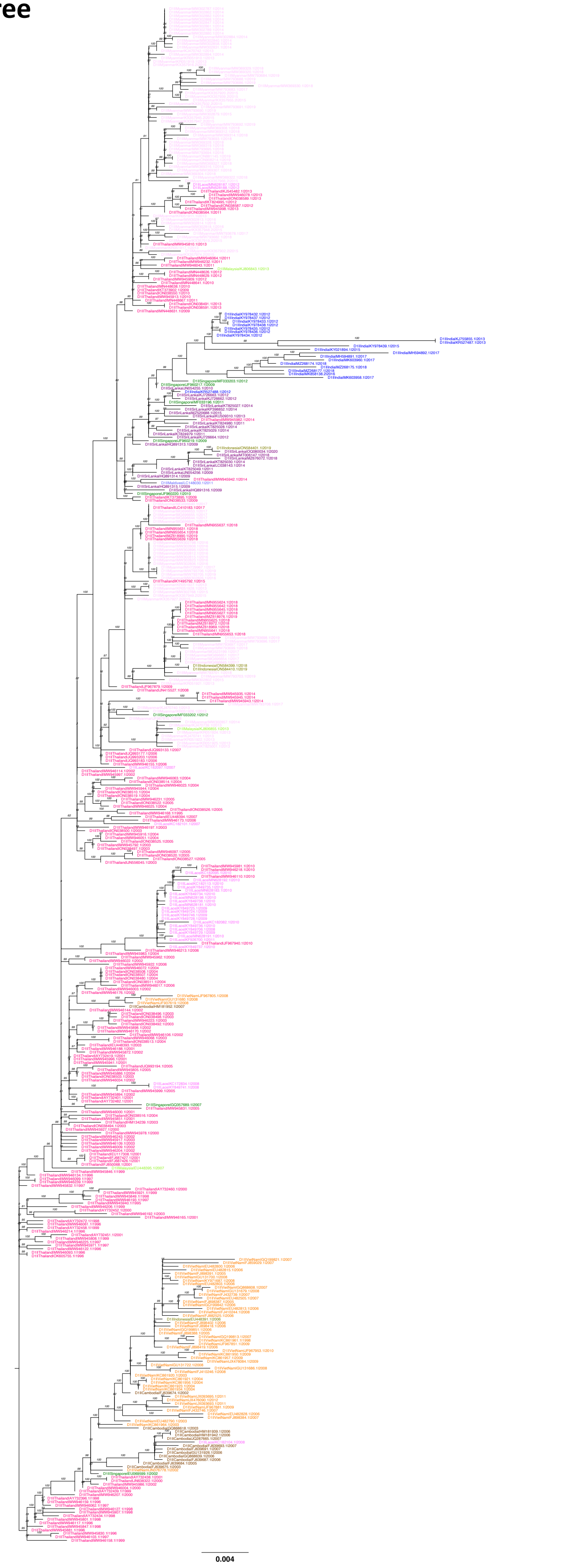

Figure S1C. DENV-1I Clade 3 (lower) subtree

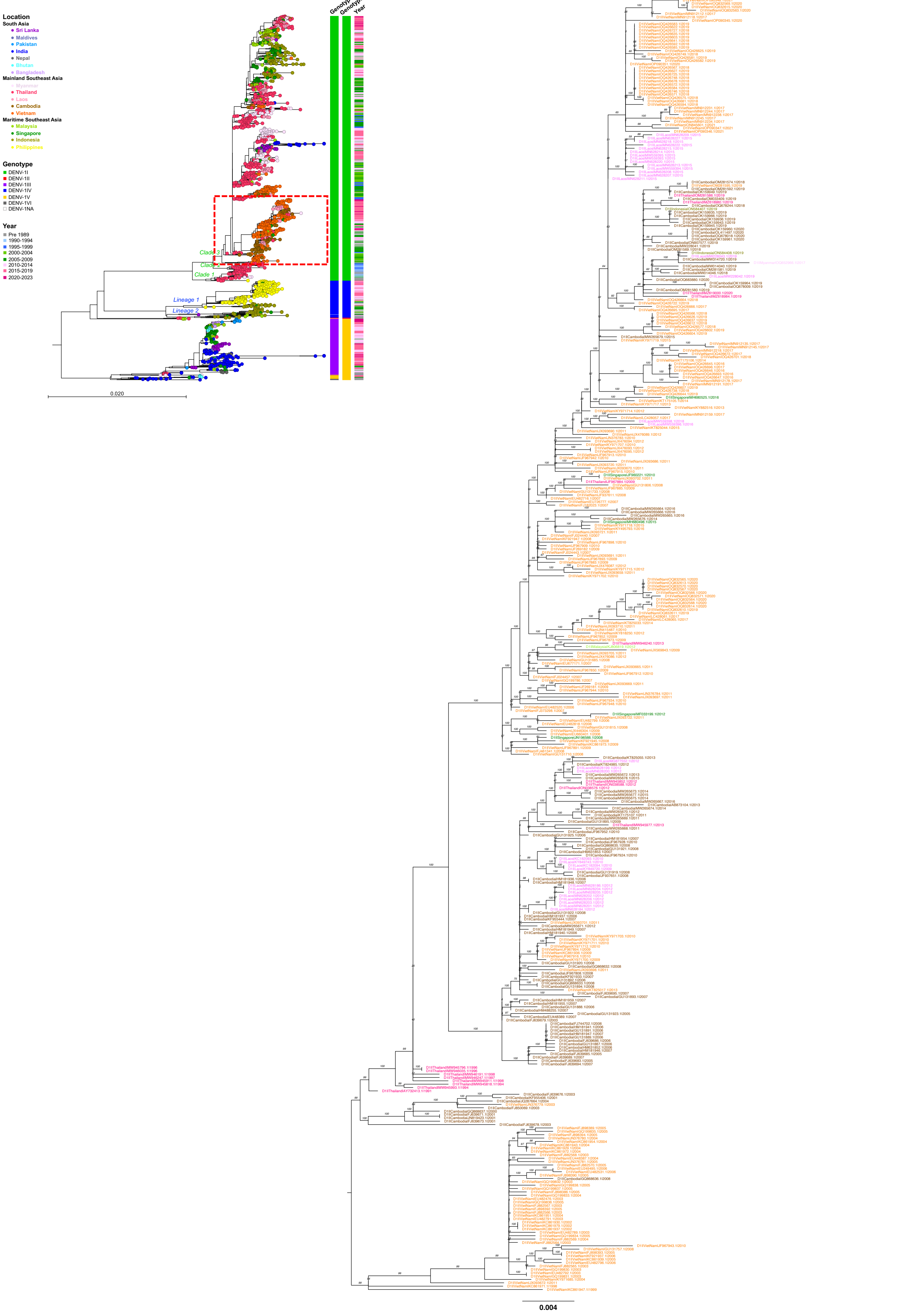

Figure S1D. DENV-1I Clade 1 and 2 subtree

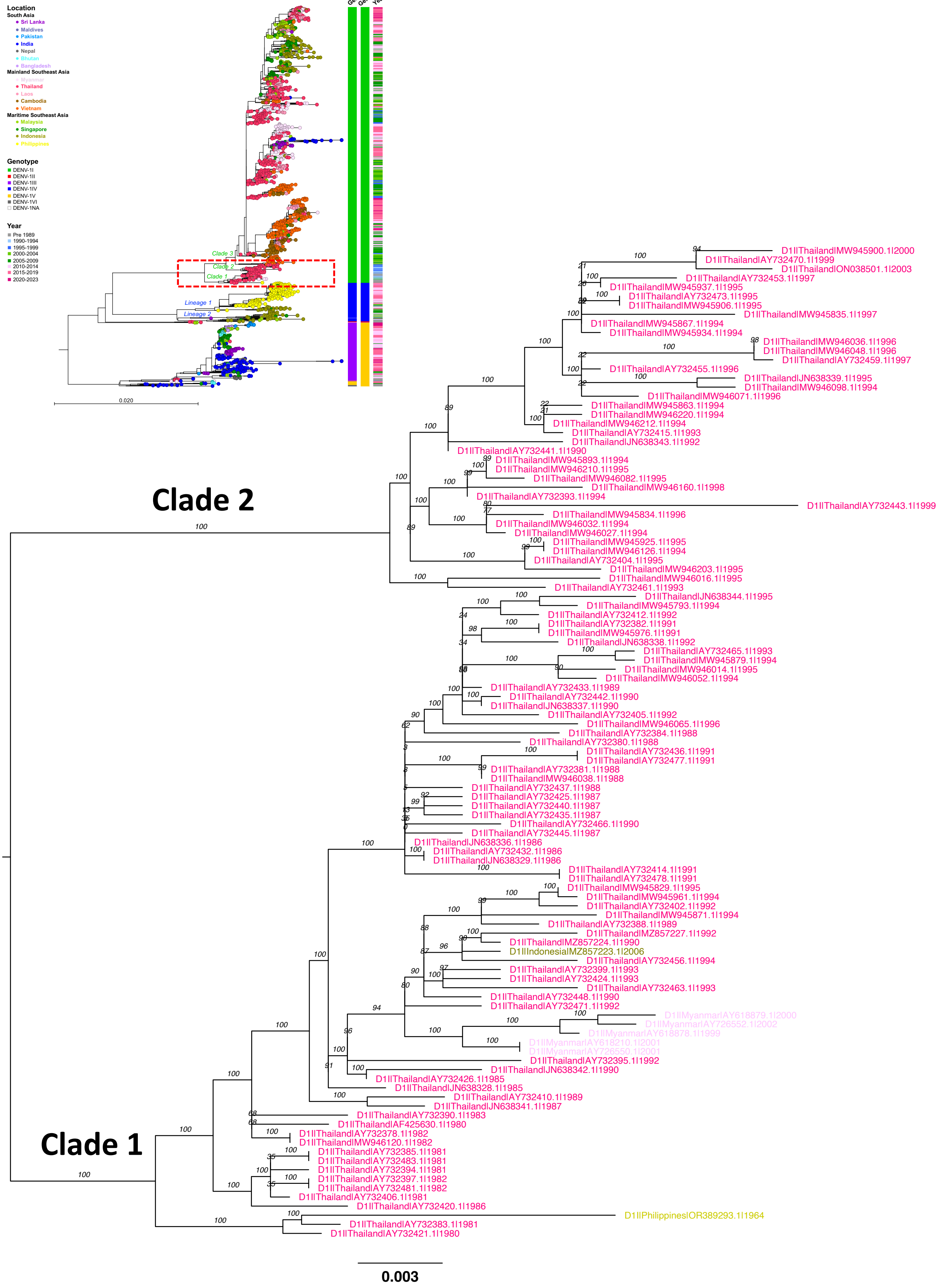

Figure S1E. DENV-1II, DENV-1IV subtree

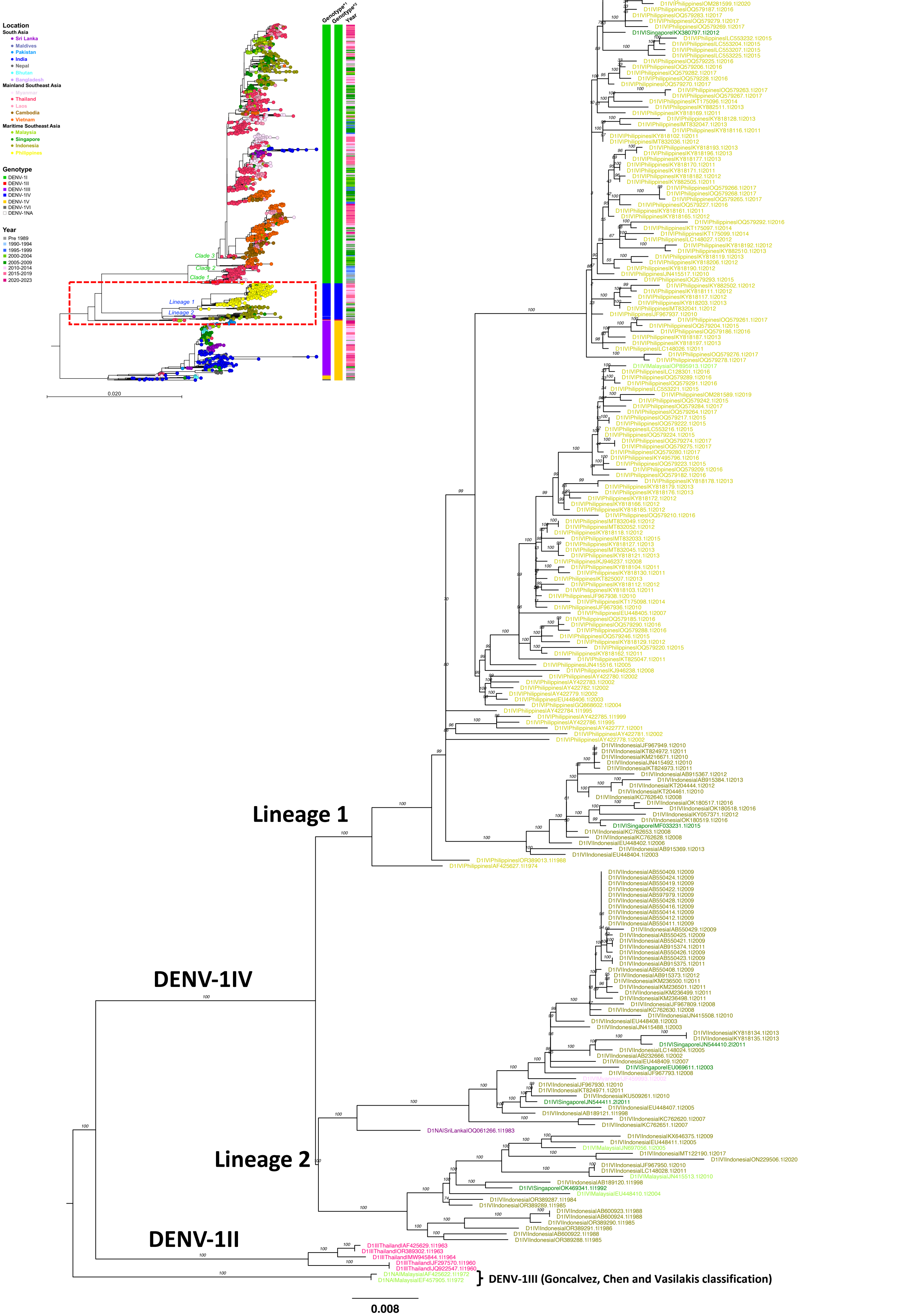

Figure S1F. DENV-1III, 1V, 1VI subtree

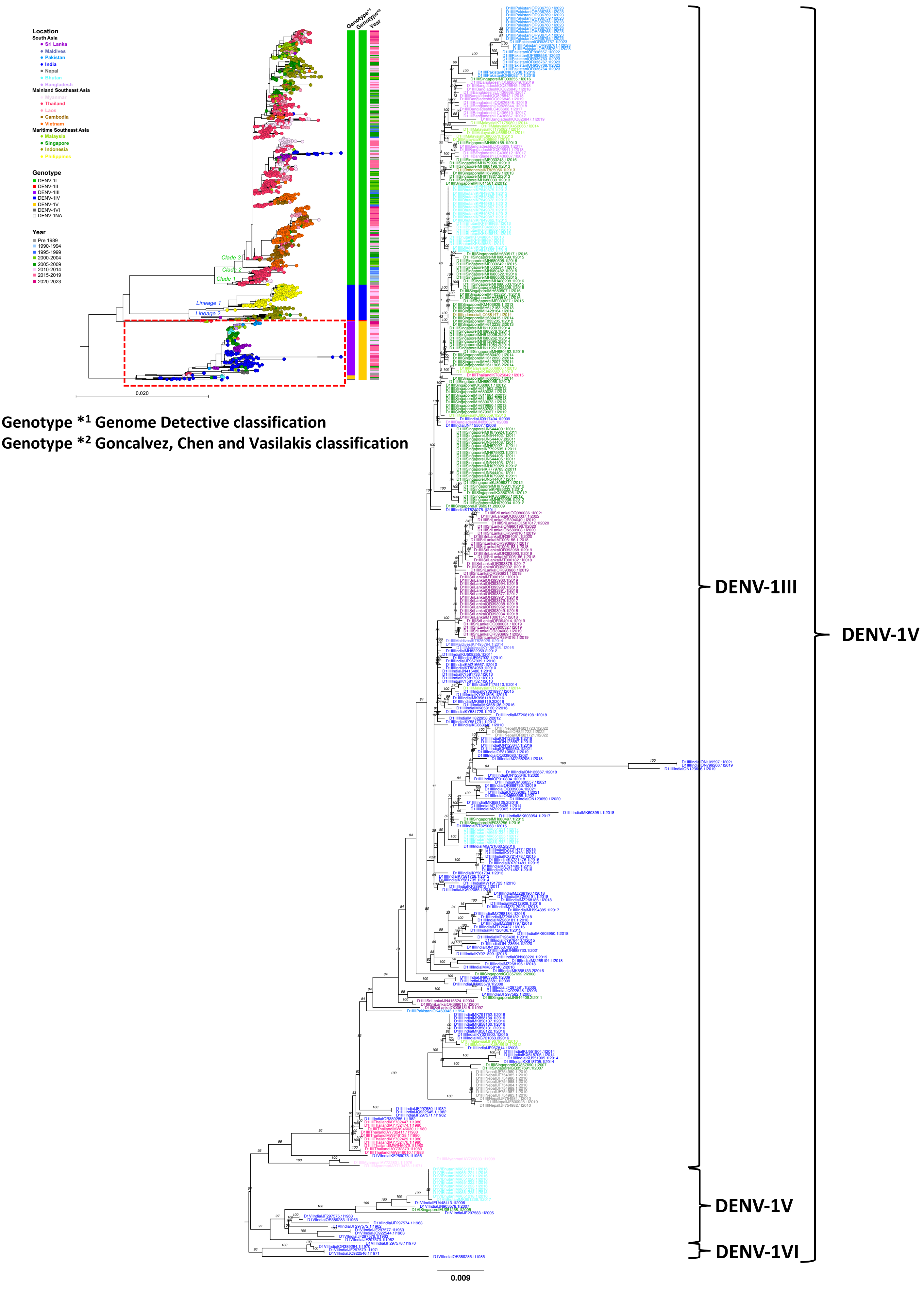

Supplement: Supplementary file 1 [file viruses-16-01046-s001.zip › Figure S1.pdf]
